# Antinociceptive Profile of Levo-tetrahydropalmatine in Acute and Chronic Pain Mice Models: Role of spinal sigma-1 receptor

**DOI:** 10.1038/srep37850

**Published:** 2016-12-02

**Authors:** Dong-Wook Kang, Ji-Young Moon, Jae-Gyun Choi, Suk-Yun Kang, Yeonhee Ryu, Jin Bong Park, Jang-Hern Lee, Hyun-Woo Kim

**Affiliations:** 1 Department of Physiology and Medical Science, College of Medicine and Brain Research Institute, Chungnam National University, Daejeon 35015, South Korea; 2 KM Fundamental Research Division Korea Institute of Oriental Medicine, Daejeon 34054, South Korea; 3 Department of Veterinary Physiology, BK21 PLUS Program for Creative Veterinary Science Research, Research Institute for Veterinary Science and College of Veterinary Medicine, Seoul National University, Seoul 08826, South Korea; 4 Department of Neuroscience and Cell Biology, University of Texas Medical Branch, Galveston, TX 77555-0625, USA

## Abstract

We have recently reported that repeated systemic treatments of extract from *Corydalis yanhusuo* alleviate neuropathic pain and levo-tetrahydropalmatine (l-THP) is one of active components from *Corydalis*. We designed this study to investigate antinociceptive effect of l-THP in acute and chronic pain models and related mechanism within the spinal cord. We found that intraperitoneal pretreatment with l-THP significantly inhibited the second phase of formalin-induced pain behavior. In addition, intrathecal as well as intraperitoneal pretreatment with l-THP reduced the mechanical allodynia (MA) induced by direct activation of sigma-1 receptor (Sig-1). In chronic constriction injury mice, these treatments remarkably suppressed the increase in MA and spinal phosphorylation of the NMDA receptor NR1 subunit expression on day 7 after surgery. Intrathecal treatment with l-THP combined with the Sig-1R antagonist, BD1047 synergistically blocked MA suggesting that l-THP modulates spinal Sig-1R activation. CatWalk gait analysis also supported that antinociceptive effect of l-THP as demonstrated by restoration of percentages of print area and single stance. Meanwhile, intrathecal pretreatment with naloxone, non-selective opioid receptor antagonist, did not affect the effect of l-THP. In conclusion, these results demonstrate that l-THP possesses antinociceptive effects through spinal Sig-1R mechanism and may be a useful analgesic in the management of neuropathic pain.

Chronic pain is a major health problem and one of the most frequent reasons for seeking medical attention^1^. The national cost of pain in the United States ranges from $560 to $635 billion, larger than the cost of the nation’s health conditions such as heart disease, cancer and diabetes^2^. People who suffer from chronic pain condition, have greater emotional distress and a reduced quality of life. Peripheral neuropathic pain, which is produced by damage or dysfunction of peripheral nerves, is characterized by mechanical allodynia (MA, pain produced in response to a non-nociceptive tactile stimulus as defined by the IASP) and thermal hyperalgesia (TH, increased sensitivity to a thermal painful stimuli). Although a variety of pathophysiologic changes are known to be involved in the neuropathic pain, the suitable analgesic drugs have a limit effect because of a wide range of side effects^3^. Therefore, the search for new analgesic compounds to serve as therapeutic alternatives is being investigated^4^.

*Corydalis tuber* (CT, a root of *Corydalis yanhusuo* W.T. Wang) is a perennial herb in the Papaveraceae family and has been used in traditional medicine on several disease^5^. Recently we have revealed that repetitive oral treatments of the extract from CT during the induction phase (day 0–5 after surgery) significantly alleviate chronic constriction injury (CCI)-induced MA, but not TH and reduce increase of spinal pNR1 in rats^6^. Levo-tetrahydropalmatine (l-THP) is one of active ingredient from *Corydalis tuber* and has an analgesic effects in several pain^7^,^8^,^9^,^10^,^11^. However, although previous reports have reported that l-THP has potential effect for treatment of inflammatory or neuropathic chronic pain, the precise mechanisms underlying the antinociceptive effect of l-THP within the spinal cord has not been established.

The development of neuropathic pain is associated with the manifestations of peripheral and central sensitization and the spinal cord is important pathway of central sensitization^12^,^13^. Spinal *N*-methyl-D-aspartate receptors (NMDARs) have been shown to play an important role in the development of central sensitization. Phosphorylation of the NR1 subunit (pNR1) at protein kinase C (PKC) and protein kinase A(PKA)-dependent sites has been demonstrated to play an important role in enhancement of NMDAR activity related to pain transmission in the spinal cord^14^,^15^. The sigma non-opioid intracellular receptor 1 (Sig-1R) has recently been recognized as a unique ligand-regulated molecular chaperone localized to the endoplasmic reticulum (ER) in cells of nervous system and has been shown to play a pronociceptive role in chronic pain models^16^,^17^. The Sig-1R translocate from the ER to the plasma membrane and they can modulate NMDAR responses^18^,^19^. We have previously reported that the direct activation of spinal Sig-1Rs using intrathecal (i.t) injection of agonist increases the response to peripheral mechanical stimuli, which is related with PKC- and PKA-dependent pNR1 in the spinal cord dorsal horn^20^,^21^. Furthermore, i.t injection of the Sig-1R antagonist attenuates the development of MA, but not TH and blocks the nerve injury induced increase of pNR1 in the spinal cord of CCI rats^22^. The antinociceptive effect of Sig-1R antagonist is similar to that of CT treatment as previously reported. In this regard, we hypothesized that l-THP may have anti-hyperalgesic effect through the spinal Sig-1R modulation in acute and chronic pain model in mice. Therefore, the present study was designed to examine: (1) whether l-THP pretreatment reduces formalin-induced pain behavior (2) the relationship with spinal Sig-1R (3) whether i.t treatment with l-THP affects MA and gate parameters in CCI mice using both with von Frey filaments and CatWalk automated quantitative gait analysis and (4) whether i.t treatment with l-THP reduces increase in spinal pNR1 expression in CCI mice.

## Results

### Effects of i.p. l-THP administration on formalin-induced nociceptive responses

**Figure 1:**
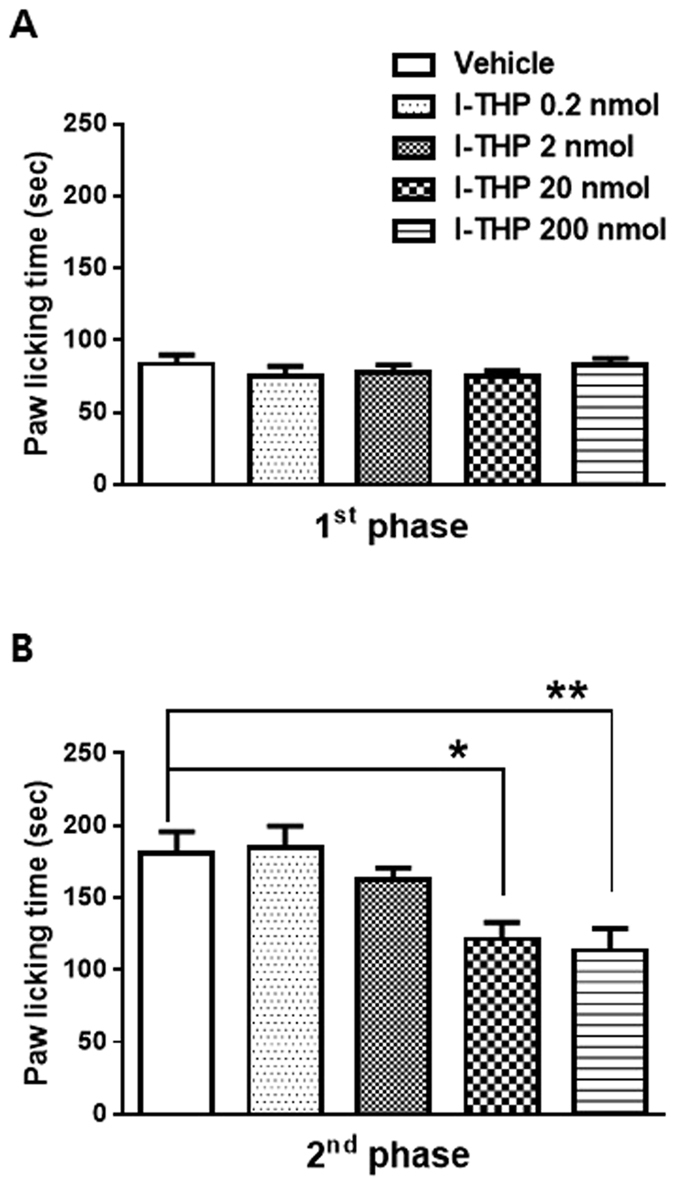
The antinociceptive effect of i.p l-THP on formalin induced pain behavior. (**A**) l-THP administration had no effect on the paw licking/biting behavior in the first phase of the formalin test. (**B**) However, the formalin induced pain behavior was significantly and dose dependently reduced by l-THP in the second phase of the formalin test as compared with control mice receiving i.p vehicle injection. (n = 8–12 per group) *P < 0.05 and **P < 0.01 as compared with that of the vehicle group.

Intraplantar (i.pl) injection of 1% formalin after vehicle pretreated mice exhibited biphasic pain behaviors during the 40 min observation period. An acute, immediate nociceptive response (licking and biting) of the injected paw lasted for 10 min (first phase: 0–10 min after formalin injection). The second phase response began after first phase and lasted for about 30 min (second phase: 10–40 min after formalin injection). Intraperitoneal (i.p) pretreatment with l-THP did not affect formalin-induced paw licking time during the first phase even the highest dose (Fig. 1A). However, pretreatment with l-THP dose-dependently suppressed the second phase of formalin-induced pain as compared with those of the vehicle-treated group (Fig. 1B; *P < 0.05 and **P < 0.01 as compared with vehicle treated group).

### Effects of l-THP administration on Sig-1R activation-induced nociceptive responses

**Figure 2:**
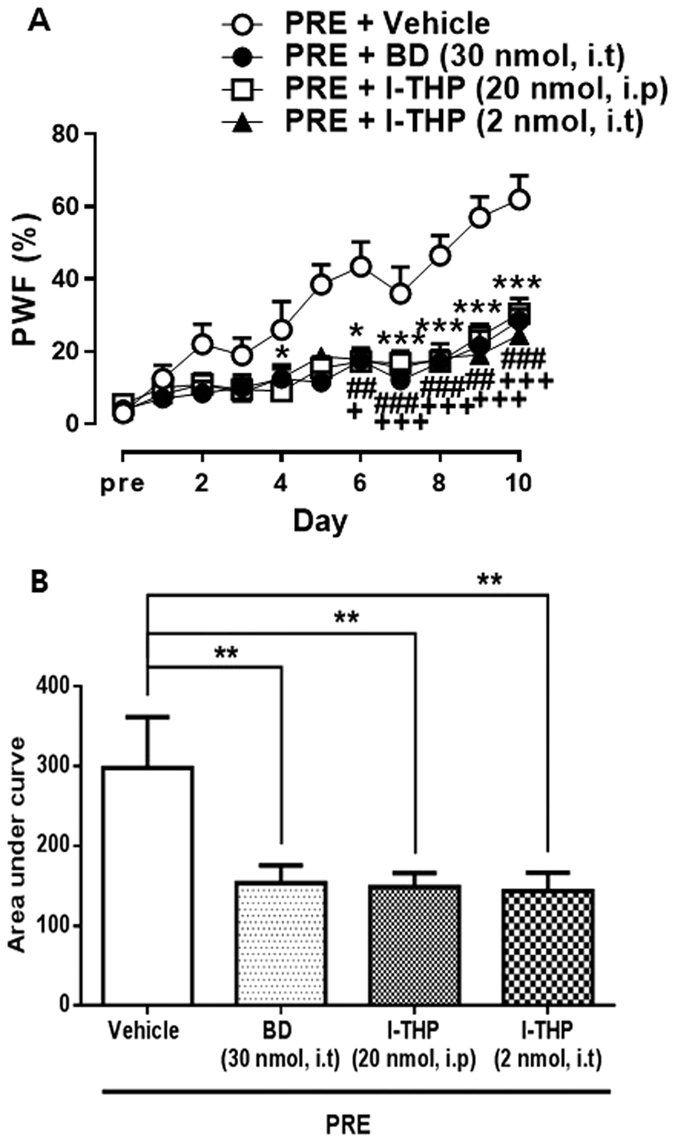
The antinociceptive effect of pretreatment with l-THP (20 nmol for i.p and 2 nmol for i.t), Sig-1R antagonist BD1047 (BD, 30 nmol, i.t) on the repeated i.t treatment of Sig-1R agonist, PRE084 (PRE)-induced changes in the paw withdrawal frequency (%). (**A**) On the time course curves. BD, *P < 0.05 and ***P < 0.001; l-THP, i.p, ##*P* < 0.01 and ###*P* < 0.001; l-THP, i.t, +*P* < 0.05 and +++*P* < 0.001 as compared with vehicle treated group. (**B**) The mean of area under curve of the Sig-1R induced mechanical allodynia was also significantly reduced by BD or l-THP pretreatment. (n = 10–11 per group) **P < 0.01 as compared with that of the vehicle group.

To evaluate the potential involvement of spinal Sig-1Rs on l-THP induced antinociceptive effect, we performed i.t. treatment with Sig-1R agonist, PRE084 (3 nmol) in naïve mice once daily for 10 days to activate spinal Sig-1R. The repeated i.t administration of the PRE084 gradually increased PWF (%) to innocuous mechanical stimuli (Fig. 2). I.t pretreatment with a Sig-1R antagonist, BD1047 (30 nmol) suppressed the PRE084-induced increase in PWF, indicating that this MA was a direct and specific result of activation of spinal Sig-1R (Fig. 2A and B; *P < 0.05 and ***P < 0.001 as compared with vehicle treated group). We next pretreated a separate group of mice with l-THP intraperitoneally prior to i.t treatment with Sig-1R agonist. As illustrated in Fig. 2, i.p pretreatment with l-THP reduced the Sig-1R induced MA compared to those injected with vehicle (##*P* < 0.01 and ###*P* < 0.001 as compared with vehicle treated group). I.t pretreated l-THP also reduced Sig-1R induced MA suggesting that this antinociceptive effect of l-THP involved spinal Sig-1R (+*P* < 0.05 and +++*P* < 0.001 as compared with vehicle treated group).

### Effects of l-THP administration on CCI-induced MA

**Figure 3:**
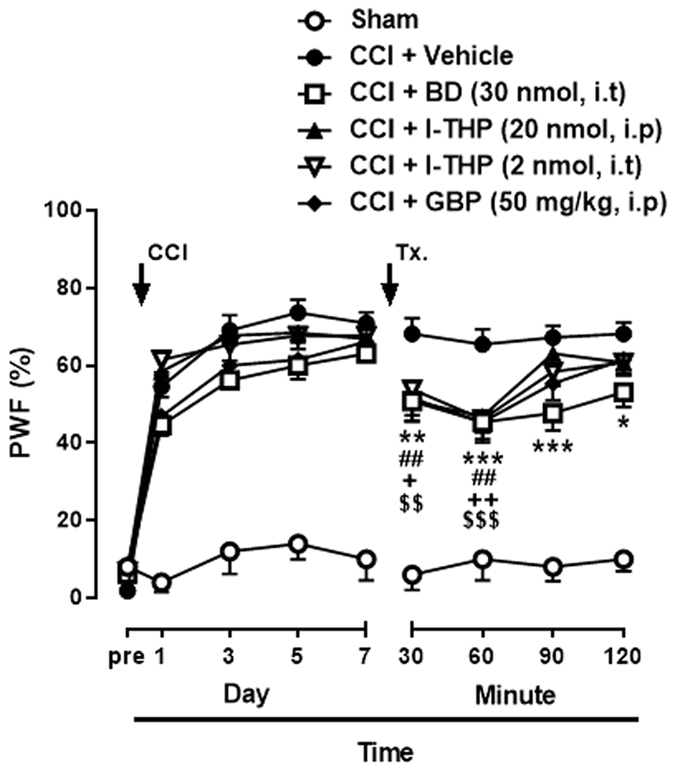
The antinociceptive effect of BD1047 (BD, 30 nmol, i.t), l-THP (20 nmol for i.p and 2 nmol for i.t), and gabapentin (GBP, 50 mg/kg, i.p) on chronic constriction injury (CCI)–induced mechanical allodynia on day 7 after CCI surgery. Gabapentin was used as a positive control drug. (n = 5 for sham group, n = 11–13 per another group) *BD, *P < 0.05, **P < 0.01 and ***P < 0.001; l-THP, i.p, ##*P* < 0.01; l-THP, i.t, +*P* < 0.05 and ++*P* < 0.01; GBP, $$*P* < 0.01 and $$$*P* < 0.001 as compared with vehicle treated group.

To investigate the potential roles of l-THP in spinal cord of chronic pain model, we examined the effect of l-THP treatment on CCI-induced MA in mice. Before CCI surgery, the baseline of PWF (%) was tested. As shown in Fig. 3, MA was developed in the ipsilateral hind paw on day 1 and maintained for 7 days after CCI surgery. We first confirmed that i.t treatment of BD1047 on postoperative day 7 significantly attenuated MA compared to that of vehicle group at 30 min after the treatment and this antinociceptive effect continued 120 min (Fig. 3; *P < 0.05, **P < 0.01 and ***P < 0.001 as compared with vehicle treated group). We next found that i.p and i.t treatment of l-THP (20 nmol for i.p and 2 nmol for i.t treatment) also led to a significant decrease in the PWF and the analgesic effect peaked 60 min after the administration as shown that of BD1047 or gabapentin treatment (##*P* < 0.01, ###*P* < 0.001 and +*P* < 0.05, ++*P* < 0.01 as compared with vehicle treated group). Gabapentin, a ligand of the α2δ1 subunit of voltage-gated calcium channels, was used to treat symptomatically as a positive drug ($$*P* < 0.01 and $$$*P* < 0.001 as compared with vehicle treated group).

### Effects of i.t. l-THP administration on CCI-induced CatWalk analysis

**Figure 4:**
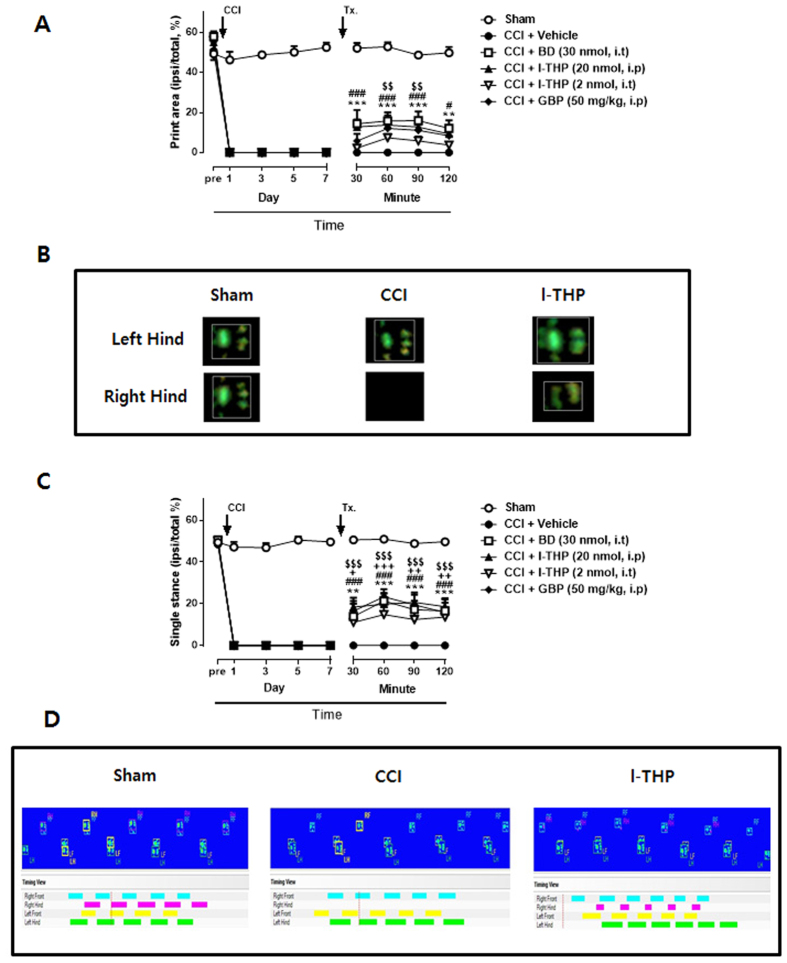
Walking track analysis in chronic constriction injury (CCI) mice. (**A**) The percentage of the ipsilateral paw print area (ipsilateral/total area %) assessed in the CatWalk analysis. Treatment of BD1047 (BD), l-THP and gabapentin (GBP) enhanced CCI-induced decrease in paw print area %. (**B**) Combined paw print image. (**C**) The percentage of the ipsilateral paw single stance (ipsilateral/total single stance %) (**D**) Representative digitized paw prints and associated step cycles. Gabapentin was used as a positive control drug. (n = 5–6 per group) *BD, **P < 0.01 and ***P < 0.001; l-THP, i.p, #*P* < 0.05 and ###*P* < 0.001; l-THP, i.t, +*P* < 0.05, ++*P* < 0.01 and +++*P* < 0.001; GBP, $$*P* < 0.01 and $$$*P* < 0.001 as compared with vehicle treated group.

CatWalk gait analysis has been used to detect gait alterations and proposed as an objective tool for evaluating sensory neuropathy induced by CCI surgery^23^,^24^. We identified two parameters which were altered significantly in the ipsi-lateral hindpaw of CCI mice. Print area was measured by calculating of surface area contacted to the glass floor. Duration of the single stance was measured in the step cycle of the hind paw where the contralateral hind paw does not touch the glass plate or where the ipsilateral hind paw touches the glass plate. The percentages of print area and single stance (ipsi-lateral/total %) was about 50% in sham surgery group throughout the 7days follow-up period (Fig. 4A and C). However, after CCI surgery, the print area and single stance of the injury paw was reduced to 0%. On day 7 after surgery, i.t treatment of BD1047 increased the percentages of both print area and single stance and these effects continued 120 min after treatment (**P < 0.01 and ***P < 0.001 as compared with vehicle treated group). I.p treatment of l-THP also increased the percentages of the injury paw as shown in Fig. 4 (#*P* < 0.05 and ###*P* < 0.001 as compared with vehicle treated group). I.t pretreatment of l-THP was shown to enhance the percentages of print area and single stance, although the effect on the print area show no significance (+*P* < 0.05, ++*P* < 0.01 and +++*P* < 0.001 as compared with vehicle treated group). The representative graph indicated that the print area and single stance in the ipsilateral paw were disappear in CCI mice because mice did not touch the glass platform (Fig. 4B and D). The print area and single stance were increased after i.t pretreatment of l-THP. The treatment of gabapentin, as a positive drug, increased the percentage of responses ($$*P* < 0.01 and $$$*P* < 0.001 as compared with vehicle treated group).

### Effects of concomitant low dose of BD1047 and l-THP treatment on the CCI-induced MA and CatWalk analysis

**Figure 5:**
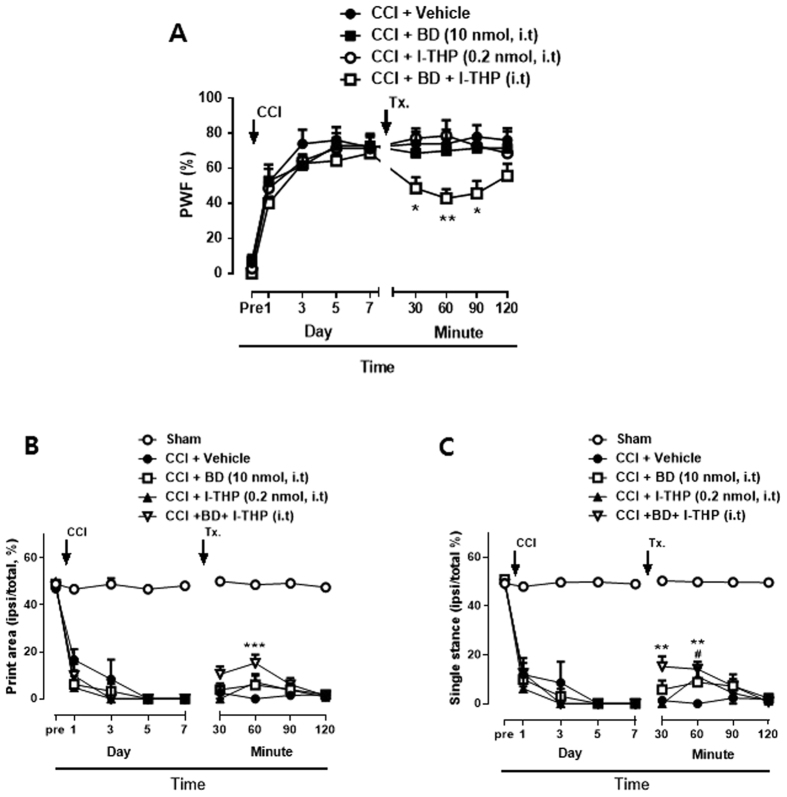
The synergic effects of concomitant l-THP and BD1047 (BD) treatment on chronic constriction injury (CCI)-induced mechanical allodynia (MA) and walking track analysis. (**A**) The low dose of l-THP or BD1047 treatment alone did not affect MA on day 7 after CCI. However, when a combination of l-THP and BD1047 was given, MA was reduced compared with the effects shown in either the l-THP or BD1047 alone treatment groups. (**B**) The low dose of l-THP or BD1047 treatment alone had no effect on the percentage of paw print area assessed in the CatWalk analysis. However, the combination of two treatments enhanced the decreased paw print area of the ipsilateral paw on day 7 after CCI. (**D**) The concomitant treatment with a low dose of l-THP and a low dose of BD1047 also showed synergic antinociceptive effect on the ipsilateral paw single stance. (n = 5–7, per group) *BD + l-THP, *P < 0.05, **P < 0.01, ***P < 0.001; #l-THP, #*P* < 0.05 as compared with those of the vehicle group.

To confirm the contribution of spinal Sig-1R activation to the antinociceptive effect of l-THP, we examined the combination effect of l-THP and BD1047 treatment. On day 7 after surgery, i.t treatment of a low dose of BD1047 (10 nmol) or a low dose of l-THP (0.2 nmol) alone did not alter CCI-induced MA. However, the combination of the two treatments (BD+l-THP) significantly decreased MA (Fig. 5A; *P < 0.05 and **P < 0.01 as compared with vehicle treated group). Similarly, the concomitant treatment with a low dose of BD1047 and a low dose of l-THP also showed synergic antinociceptive effect on CatWalk analysis. The low dose of l-THP slightly increased the percentages of single stance of injury paw on 60 min after the treatment (Fig. 5C; #*P* < 0.05 as compared with vehicle treated group). However, the combination of the two treatments (BD + l-THP) significantly increased the percentages of print area and single stance from 30 min after the injection and sustained for 60 min (Fig. 5B and C; **P < 0.01 and ***P < 0.001 as compared with vehicle treated group). These results suggest that a significant relationship between l-THP and spinal Sig-1Rs activation.

### Effects of i.t. l-THP administration on spinal pNR1 expression in CCI mice

**Figure 6:**
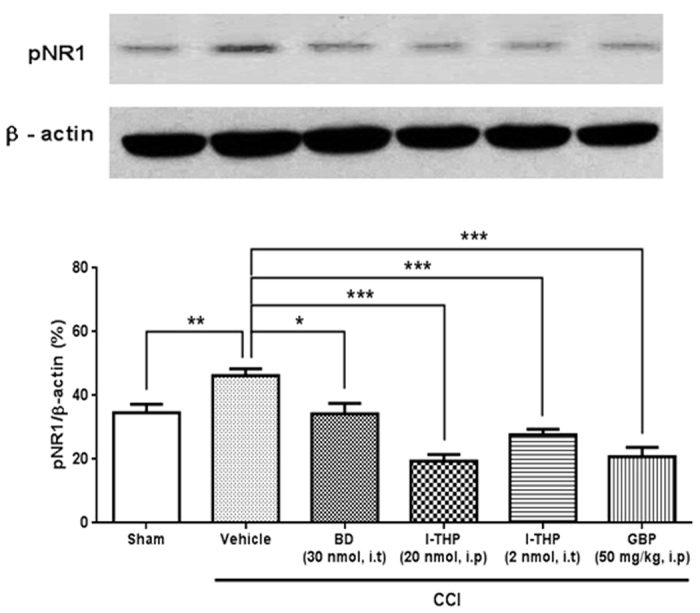
Inhibitory effect of l-THP on chronic constriction injury (CCI)-induced pNR1 expression. As compared with sham group, CCI remarkably increased the phosphorylation of NMDA NR1 subunit (pNR1) expression. Treatment of BD1047 (BD), l-THP and gabapentin (GBP) significantly reduced the pNR1 expression in ipsilateral dorsal horn as compared with that of vehicle group. (n = 5 per group) *P < 0.05 and ***P < 0.001 as compared with those of the vehicle group.

Mice were killed at 120 min after behavioral measurement of each drug treatment on day 7 after CCI surgery. Sham mice were killed at the same time points for comparison. Western blot analysis was performed to examine whether the CCI-induced increase in pNR1 expression was regulated by l-THP treatment. As shown in Fig. 6, the expression of pNR1 in the ipsilateral spinal cord dorsal horn was significantly increased on day 7 after CCI surgery (**P < 0.01 as compared with sham group). This increase was reduced by i.t pretreatment of BD1047. We confirmed that both i.p and i.t treatment with l-THP were significantly reduced CCI-induced increase in pNR1 expression as much as the effect of BD1047 or gabapentin (*P < 0.05, **P < 0.01 and ***P < 0.001 as compared with vehicle treated group).

### No relation with opioid R

**Figure 7:**
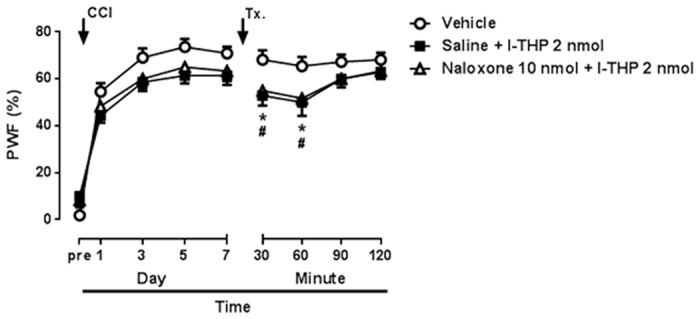
The effect of treatment of naloxone (10 nmol, i.t) on chronic constriction injury (CCI)-induced MA on day 7 after CCI surgery. Non-selective opioid receptor antagonist, naloxone did not influence the antinociceptive effect of l-THP treatment in the CCI model. (n = 7–11 per group).

To examine the involvement of opioid receptor on antinociceptive effect of l-THP, we intrathecally treated a dose of naloxone (non-selective opioid receptor antagonist, 10 nmol) in combination with 2 nmol l-THP (Fig. 7). The naloxone treatment did not affect the antinociceptive effect of i.t treatment l-THP suggesting that opioid receptor does not related with the antinociceptive mechanism of l-THP in the spinal cord.

### Rota-Rod Test

**Figure 8:**
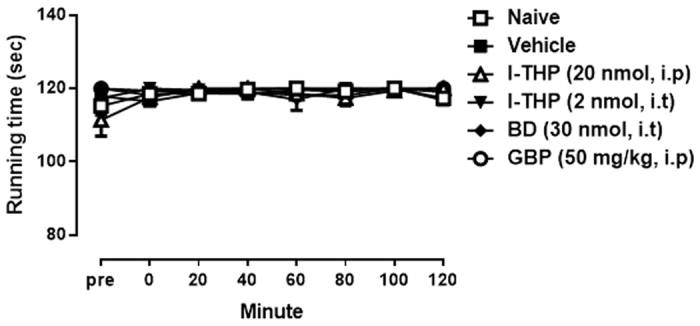
Effects of the drugs used in this study on motor function. Rota-rod test revealed that the treatments of l-THP (20 nmol for i.p and 2 nmol for i.t), BD1047 (BD, 10nmol, i.t) and gabapentin (GBP, 50 mg.kg, i.p) did not affect normal motor function on 120 min after treatment as compared with that of naïve mice or vehicle treated group. (n = 5–8 per group).

The effect of l-THP on motor function was examined using the rota-rod test. Naïve mice remained on the rotarod apparatus about 120 sec. After drugs treatments, each animal was tested on the rotarod apparatus and the time for which the mouse was able to remain on the bar was recorded. I.t and i.p treatments of l-THP, i.t treatment of BD1047 and i.p treatment of gabapentin did not affect normal function of mice on 120 min after treatment and produced no significant change in the rotarod latency (Fig. 8). This result indicated that antinociceptive effects of l-THP, BD1047 and gabapentin are not induced by motor impairment.

## Discussion

l-THP has been used clinically in china for more than 40 years as an analgesic drug for the treatment of mild or moderate pain. Recently, it has been reported the analgesic effect of systemic administration with l-THP in several pain models. Cao *et al*. reported that intragastric pretreatment of dl-THP suppress persistent spontaneous nociception induced by i.p injection of acetic acid in visceral pain model^8^. They also reported that dl-THP pretreatment of the higher dose produce distinct suppression of bee venom or formalin induced persistent nociception. In addition, i.p of l-THP treatment produces a dose-dependent anti-hyperalgesic effect in a mouse model of chemotherapeutic agent oxaliplatin-induced neuropathic pain^9^. Furthermore, systemic treatment with l-THP exerts remarkable antihyperalgesic effects in a dose dependent manner in a neuropathic pain induced by segmental spinal nerve ligation and inflammatory pain induced by an intraplantar injection of complete Freund’s adjuvant^10^. Although previous studies have suggested that the l-THP has a great antinociceptive effect, the precise mechanism of l-THP in the spinal cord has not been elucidated.

The present study demonstrated for the first time that the antinociceptive mechanism of l-THP within the spinal cord using acute and chronic pain model in mice. First, i.p administration of l-THP significantly reduced formalin-induced nociceptive behavior in the second phase of the formalin test. The formalin test is persistent ongoing pain model, which is characterized by biphasic nociceptive responses consisting of immediate (the first) and latent (the second) phases. The first phase response of the formalin test is caused predominantly by the direct stimulation of nociceptors due to the peripheral stimulus and is thought to be an acute pain reaction, while the second phase appears to be dependent on the combination of an inflammatory reaction in the peripheral tissue and functional changes in the dorsal horn of the spinal cord^25^. Our results showed that l-THP impacts on the mechanism associated with the spinal functional changes rather than the peripheral sensitization.

In addition, i.t pretreatment with l-THP was shown to block the development of MA induced by repeated spinal Sig-1R activation, implicating the antinociceptive mechanism of l-THP in *in-vivo* animal model. As supporting this data, additional *in-vitro* study such as an examination of receptor affinity between l-THP and Sig-1R may help to show the potential efficacy and therapeutic usage of l-THP on pain diseases. Repeated daily i.t injection of PRE084 gradually increase the nociceptive response and this increase was blocked by pretreatment with the Sig-1R antagonist, BD1047. It is consistent with the study that chronic intracisternal infusion of Sig-1R agonist time-dependently facilitate neuronal activity, including Fos elevation associated with migraine-like pain behavior^26^. In a chronic neuropathic pain model, i.t treatment with l-THP, as well as the Sig-1R antagonist BD1047, significantly reduced the MA and pNR1 expression on day 7 after CCI surgery. Interestingly, low doses of BD1047 produced synergic effect on MA when combined with low doses of l-THP. These results indicate that l-THP can modulate Sig-1R activation and contribute to MA in neuropathic mice.

CatWalk analysis also supported the antinociceptive effect of the treatment. In CatWalk analysis, the treatment of l-THP as well as combined treatment with BD1047 increased the percentages of both print area and single stance and these effects continued 120 min after treatment. CatWalk gait analysis detects dynamic and static gait parameters including base of support, stride length, maximum area, print area, mean intensity, stance duration, swing duration, regularity index and phase lags. Vrinten *et al*. reported that rats suffering from CCI of the sciatic nerve demonstrated alterations of gait parameters that correlated closely with mechanical withdrawal thresholds obtained by the von Frey method^23^. Furthermore, the CatWalk XT system detected subtle neurobehavior alterations in intensity of nerve damage, even though these changes were not determined by MA and TH^24^. Hence, the CatWalk system seems to be a useful test for detecting the severity of neuropathic pain. In our knowledge, no previous study has been reported that anti-nociceptive effects of l-THP or Sig-1R antagonist on neuropathic pain model using this technique.

The role of Sig-1R in modulating central sensitization associated with chronic pain has recently been investigated. The blockade of spinal Sig-1Rs using i.t injection of Sig-1R antagonist BD1047 or using Sig-1R knockout mice reduces the spontaneous pain behavior with formalin test^27^,^28^. De la Puente *et al*. reported that Sig-1R knockout mice does not show cold and MA and does not exhibit increased phosphorylation of ERK in the spinal cord after sciatic nerve injury^29^. These reports are consistent with our data showing that i.t injection of Sig-1R antagonist attenuated MA and increase of pNR1 when administered during the induction phase^22^. NMDA receptor activation is known to be an essential contributor to the process of central sensitization, particularly to the early phosphorylation-dependent phase of central sensitization^14^,^15^. Recently, several reports have suggested the modulating mechanism of Sig-1R on NMDA receptor activation via direct or indirect pathway. Pabba *et al*. reported that Sig-1R activation leads to an increased in the expression of GluN2 subunits, as well as trafficking of NMDARs to the cell surface in the rat hippocampus^19^. In addition, it has also been reported that high concentrations of Sig-1R ligands potentiate NMDAR responses by down-regulating small conductance calcium-activated K+(SK) channels^18^. Furthermore, we recently reported that spinal Sig-1R localize to astrocyte and activated Sig-1R increases the production of NMDA receptor co-agonist, D-serine^30^. Although, it is unclear that how Sig-1R activation modulates pNR1 expression induced by l-THP treatment condition, these results suggest that the antinociceptive effect of l-THP in the spinal cord was primarily mediated by Sig-1R activation relating with increase of pNR1.

It has been suggested that low concentrations of corydalis reduced glycine-induced ion current in PAG neurons and the inhibitory action was partially abolished by treatment with naltrexone, a non-selective opioid antagonist^31^. In addition, intra periaqueductal gray (PAG), one of the descending brainstem pain modulation system, injection of naloxone markedly attenuate the analgesic action of l-THP^32^. However, in the present study, i.t pretreatment with naloxone did not affect the antinociceptive effect of l-THP indicating that opioid receptor is not involved in mechanism of l-THP in the spinal cord. Meanwhile, Hu *et al*. reported that the analgesic action of i.p treatment with l-THP is mediated by blocking supraspinal dopamine D2 receptors^33^. Recently, in a chronic pain model such as chemotherapy induced pain model or nerve injury model, i.p treatment with l-THP produced a dose-dependent anti-hyperalgesic effect and this effect was partially blocked by a systemic administration of dopamine D1 receptor antagonist, suggesting a dopamine D1 receptor mechanism^9^,^10^. Therefore, it is possible that the effect of l-THP is mediated by interaction in supraspinal or spinal dopamine receptor and spinal Sig-1R.

In conclusion, the present study shows for the first time that i.t treatment of l-THP has a potent anti-allodynic effect in neuropathic pain mice without motor impairment. This antinociceptive mechanism may involve spinal sigma-1 receptor activation, relating to pNR1 expession. The current study improves the understanding of mechanisms of l-THP induced antinociception and provides scientific basis for the clinical application of the l-THP treatment.

## Materials and Methods

### Animals

Male ICR mice (Samtako, Osan, South Korea) weighing 20–25 g were used. All experiments were conducted in accordance with the ethical guidelines of the International Association for the Study of Pain and were approved by the Animal Care and Use Committee at the Chungnam National University. Food and water were supplied ad libitum and animals were kept in a 12-hour light/dark cycle, a constant room temperature (maintained between 20 and 25 °C), and 40–60% humidity.

### Drug administration

The following drugs were used: Levo-tetrahydropalmatine (l-THP; Santa Cruz Biotechnology, Santa Cruz, CA, USA); PRE084 (a selective sigma-1 receptor agonist, Sigma); BD1047 (a selective sigma-1 receptor antagonist, Sigma); gabapentin (GBP, a ligand of the α2δ1 subunit of voltage-gated calcium channels, Fluka); naloxone (a non-selective opioid receptor antagonist, Sigma). The doses used in the present study were selected based on doses previously used in literatures^28^,^34^,^35^,^36^. The drugs were dissolved in 10 μl of saline, while l-THP was dissolved in 5% dimethyl sulfoxide (DMSO, Sigma, St. Louis, MO, USA) in saline solution. For i.t injection, we used the modified method of direct transcutaneous i.t injection in mice^37^. I.t injections were made into the L5-L6 intervertebral space using a 50 μl Hamilton syringe connected to a 30-gauge needle. The flick of the tail was considered indicative of a successful i.t administration.

### Formalin Test

The formalin test was carried out as previously described^28^. Before formalin test, mice were acclimated for 30 min in an observation chamber. Formalin (1%, 20 μl) was injected subcutaneously into the plantar surface of the right hind paw with a 30 gauge needle. Following formalin injection, the animals were back to the plexiglass observation chamber and behavioral responses were recorded using a video recording system. The summation of time (in seconds) spent paw licking and biting time was measured at every 5 min time block during 40 min post-injection period. Behavior assessment of recorded video files was performed by an observer blinded to the treatment condition. Paw licking and biting time was divided into first (0–10 min post-injection) and second (10–40 min post-injection) phases for the further analysis. Intraperitoneal (i.p) pretreatment with l-THP (0.2, 2, 20 or 200 nmol) in a volume of 0.1 ml was performed 30 min prior to formalin injection. Vehicle group mice received 5% DMSO solution in saline.

### Mechanical allodynia assay

To assess nociceptive responses to innocuous mechanical stimuli (mechanical allodynia), we measured paw withdrawal response frequency (PWF) by using von Frey filament (2.0 g, North Coast Medical, Morgan Hill, CA, USA) as describe previously^38^. A von Frey filament was applied from underneath the metal mesh flooring to each plantar of hind paw. The filament was applied 10 times to each paw, with 10 sec of time interval. The number of paw withdrawal responses after each filament stimulus was then counted and results were expressed as percentage withdrawal response frequency (PWF, %).

### CCI-Induced Neuropathic Pain

A CCI of the common sciatic nerve was performed according to the method described by Bennett and Xie^39^ with minor modification. After obtaining normal baseline values of PWF on 1 day before surgery, mice were randomly assigned to experimental and control groups. Animals were anaesthetized by i.p injection with Zoeltil^®^ (1.6 mg/kg, Virbac Korea, Seoul, South Korea) and Rompun^®^ (0.8 mg/kg xylazine HCl, Bayer Korea, Seoul, South Korea,). The right sciatic nerve was surgically exposed at the mid-thigh level, and 3 loose ligatures of 4–0 chromic gut were placed around the nerve with a 1.0 to 1.5 mm interval between each ligature. Sham mice received surgical opening in the same manner without nerve ligation. After surgery, animals were placed on the heating pad until the recovery from anesthesia. Postoperative MA were measured on 1, 3, 5, 7 days post-surgery. On day 7 after CCI surgery, mice received vehicle (5% DMSO, i.p), BD1047 (30 nmol, i.t.), l-THP (20 nmol for i.p. and 2 nmol for i.t.) or GBP (50 mg/kg, i.p.). After administration of each drug, PWF (%) was measured as described above at every 30 min for 120 min. For mechanism test, i.t. treatment with naloxone (10 nmol, i.t) was performed 30 min prior to i.t. injection of l-THP on a day 7 after CCI surgery. All behavioral analysis were performed blindly.

### CatWalk Automated Gait Analysis in CCI Mice

The CatWalk XT system (Noldus Information Technology, Netherlands) was used to evaluate the gait parameter in several pain models such as osteoarthritis or neuropathic pain^24^,^40^. During mice walks freely through dark tunnel, downward pressure of each paw illuminates fluorescent light from the glass platform and video camera detects and records these light signals. Then CatWalk XT software analyzes automatically various gait parameters such as paw print area, duty cycle, stance phase and so on. In this study, we examined two parameters (print area and single stance) to determine the antinociceptive effect of l-THP on CCI mice. Print area was measured by calculating of surface area contacted to the glass floor and single stance is the part in the step cycle of the hind paw where the contralateral hind paw does not touch the glass plate or where the ipsilateral hind paw touches the glass plate. All CatWalk data was gained by calculating of percent change between ipsi- and contra-lateral hind paws (i.e. normal mice showed about 50% which means data of ipsi vs. contra was 50 to 50).

### Western Blot Analysis of pNR1 in CCI Mice

As following the method of our previous report^20^, we examined the inhibitory effect of l-THP on CCI-induced pNR1 in spinal dorsal horn. Mice were anesthetized at 120 min after each drug treatment on day 7 after CCI surgery. Then the ipsilateral dorsal quadrat from each mouse was collected and snap-frozen in liquid nitrogen. Lysates of the spinal cord (25 μg) were separated by 9% SDS-PAGE and then transferred to a nitrocellulose membrane. Nonspecific binding was blocked with 5% bovine serum albumin (MP Biomedical, Auckland, New Zealand) for 1 hour at room temperature. The membrane was then incubated with 1:1000 dilution of anti-phospho NMDA receptor 1 antibody (Abcam, Cambridge, UK) and 1:4000 dilution of anti-β-actin antibody (Calbiochem, Darmstadt, Germany) in the blocking solution for overnight at 4 °C. The membrane was washed and incubated with 1:2000 dilution of horseradish peroxidase-conjugated anti-rabbit antibody (Calbiochem, Darmstadt, Germany) for 1 hour at room temperature. After washing five times with TBST, antibody-reactive proteins were detected using a chemiluminescence assay kit (Pharmacia-Amersham, Freiburg, Germany). The positive pixel area of specific bands was measured with a computer-assisted image analysis system and normalized against the corresponding β-actin loading control bands.

### Rota-Rod Test

Rota-rod test is a commonly used screening procedure to examine motor incoordination and/or ataxia in rodents. In addition, this test can differentiate antinociceptive effect of drug from adverse side effect such as sedation. As following the method described previously^38^, mice were placed on a cylindrical platform (12 cm wide; 3 cm diameter) suspended 33 cm above the bottom of the apparatus (SciTech Korea Inc., Seoul, Korea). Falls were cushioned by wood shaving bedding. After 3 days of acclimation period, rota-rod test has done before the administration and at every 20, 40, 60, 80, 100, and 120 min after the administration of vehicle, l-THP (2 nmol for i.t. and 20 nmol for i.p.), BD1047 (30 nmol, i.t) and gabapentin (50 mg/kg, i.p). Time spent on a rotating rod (constant speed of 5–6 revolutions per min) was measured at each time point and cut-off time was 2 min.

### Statistical Analysis

Statistical analysis was performed using Prism 5.0 (GraphPad Software Inc., La Jolla, CA, U0SA). One-way ANOVA was performed to determine the effect of drugs on formalin induced pain behavior. Repeated measures of two-way analysis of variance (ANOVA) was performed to determine overall effects in the time-course of other nociceptive behavioral tests. Post-hoc analysis was performed using the Dunnett’s test in order to determine the *P* value among the experimental groups. For western blot analysis, band intensity was quantified in a Epichem 3 Darkroom using LabWorks software (UVP, Upland, CA, USA) followed by normalization to the density of β-actin, and column analysis was used by Student’s t-test for comparisons between the two mean. A value of *P* < 0.05 was considered statistically significant.

## Additional Information

**How to cite this article**: Kang, D.-W. *et al*. Antinociceptive Profile of Levo-tetrahydropalmatine in Acute and Chronic Pain Mice Models: Role of spinal sigma-1 receptor. *Sci. Rep.* **6**, 37850; doi: 10.1038/srep37850 (2016).

**Publisher's note:** Springer Nature remains neutral with regard to jurisdictional claims in published maps and institutional affiliations.
